# Ten quick tips for avoiding pitfalls in multi-omics data integration analyses

**DOI:** 10.1371/journal.pcbi.1011224

**Published:** 2023-07-06

**Authors:** Davide Chicco, Fabio Cumbo, Claudio Angione

**Affiliations:** 1 Institute of Health Policy Management and Evaluation, University of Toronto, Toronto, Ontario, Canada; 2 Genomic Medicine Institute, Lerner Research Institute, Cleveland Clinic, Cleveland, Ohio, United States of America; 3 School of Computing Engineering and Digital Technologies, Teesside University, Middlesbrough, United Kingdom; McGill University, CANADA

## Abstract

Data are the most important elements of bioinformatics: Computational analysis of bioinformatics data, in fact, can help researchers infer new knowledge about biology, chemistry, biophysics, and sometimes even medicine, influencing treatments and therapies for patients. Bioinformatics and high-throughput biological data coming from different sources can even be more helpful, because each of these different data chunks can provide alternative, complementary information about a specific biological phenomenon, similar to multiple photos of the same subject taken from different angles. In this context, the integration of bioinformatics and high-throughput biological data gets a pivotal role in running a successful bioinformatics study. In the last decades, data originating from proteomics, metabolomics, metagenomics, phenomics, transcriptomics, and epigenomics have been labelled *-omics* data, as a unique name to refer to them, and the integration of these omics data has gained importance in all biological areas. Even if this omics data integration is useful and relevant, due to its heterogeneity, it is not uncommon to make mistakes during the integration phases. We therefore decided to present these ten quick tips to perform an omics data integration correctly, avoiding common mistakes we experienced or noticed in published studies in the past. Even if we designed our ten guidelines for beginners, by using a simple language that (we hope) can be understood by anyone, we believe our ten recommendations should be taken into account by all the bioinformaticians performing omics data integration, including experts.

## Introduction

Integration of omics data is a pillar of bioinformatics: Incorporating data of genomics, proteomics, metabolomics, metagenomics, phenomics, transcriptomics, epigenomics, and other -omics areas in a unique database, in fact, can provide a larger picture of a specific biological aspect and, therefore, facilitate the discovery of more relevant, interesting, and solid scientific results. Just like in photography, where photos of the same subject taken from different angles can provide different perspectives and pieces of information about the same phenomenon, bioinformatics data chunks of different types and coming from different sources can be more informative than a single-source dataset.

Multi-omics data integration, however, can bring several problems, especially if performed by beginners or apprentice researchers. For example, apprentice bioinformaticians sometimes produce multi-omics resources based solely on their perspective, without taking into account what the analysts would really need. The final database then becomes difficult to use and gets underutilized in the bioinformatics community, possibly generating a waste of effort, time, energy, and funds. Or, sometimes, apprentice bioinformaticians do not give enough importance to the metadata, only realizing too late that their biological data do not have enough descriptive metadata to be used broadly by the scientific community. To avoid these and other pitfalls and common mistakes, we propose this study where we describe a few guidelines to keep in mind when performing multi-omics data integration. We designed these quick tips for data curators, biomedical data scientists, machine learning analysts, computational biologists, bioinformaticians, and students who are going to perform a multi-omics data integration phase to produce an omics data resource to be used by analysts.

The potentials and advantages of multi-omics data integration were described in several studies. Sijia Huang and colleagues [1], for example, reported the recent progress in the field by explaining the most common and successful techniques for this scope. A more recent article by Indhupriya Subramanian and colleagues [2], on another hand, provided a detailed and thorough overview of the multi-omics general situation. Examples of effective multi-omics integration tools are mixOmics [3] in R and INTEGRATE [4] in Python. Sebastian Canzler and coauthors [5] proposed some perspective recommendations and challenges related to toxicology, while Mingon Kang and colleagues [6] proposed a roadmap for omics integration using deep learning.

**Fig 1:**
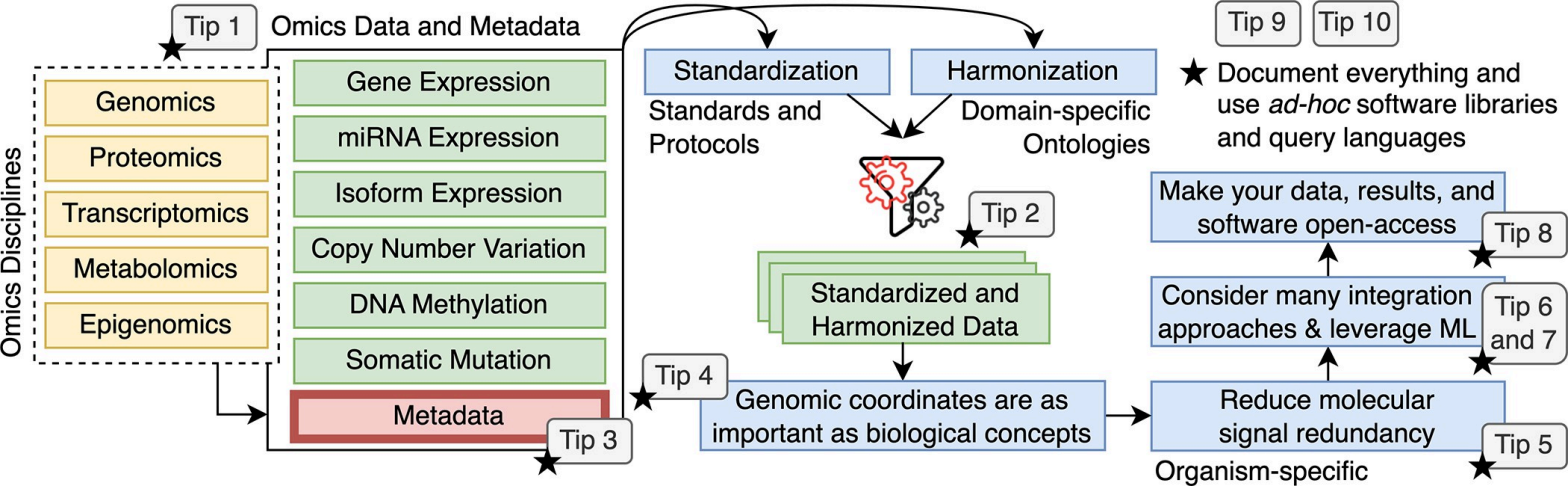
Schematization of the proposed tips as a flow chart that shows all the steps we suggest to follow in order to facilitate the integration and analysis of multi-omics data. Every tip, from the first up to the seventh, are marked with a black star, meaning that we suggest documenting everything and using ad hoc software libraries and query languages as suggested in Tip 9 and Tip 10.

In the *PLOS Computational Biology* education collection, no study provided recommendations for bioinformatics data integration so far. A study by Ramon Diaz-Uriarte and colleagues [7] proposed ten quick tips for biomarker discovery and its validation that, although interesting, does not regard multi-omics data integration directly. We fill this gap by presenting our current quick tips for multi-omics data integration as summarized in Fig 1.

Several studies follow the same pipeline described here while dealing with multi-omics data. A striking example is the research study by Eleonora Cappelli and colleagues [8] whose aim is to combine DNA methylation and RNA sequencing data that the authors used to train and test a supervised classification model for identifying disease-specific biomarker genes. In particular, they focused their analysis on three different types of cancer: breast invasive carcinoma (BRCA), thyroid carcinoma (THCA), and kidney renal papillary cell carcinoma (KIRP). The authors retrieved the data of these cancer types from the TCGA2BED [9] database, which provides all the publicly available data of the TCGA program standardized into the free-BED format. The authors integrated DNA methylation data with the beta values of the methylated CpG island among with RNA sequencing data with the expression of genes, by joining data based on common genomic coordinates. That is, if the RNA sequencing data contains the expression of specific genomic regions that refer to the genes, the specific single-nucleotide positions in the genomic regions of the genes can be methylated. The authors eventually analysed these data with different tree- and rule-based supervised classification algorithms (for example, C4.5 [10], Random Forests [11], RIPPER [12], and CAMUR [13]) producing over 15,000 classification models (in the form of gene sets) able to discriminate case and control samples with an accuracy of 95% on average. Most importantly, the authors documented every step of their analysis and made their software code openly available along with their results.

### Tip 1: Design the integrated data resource from the perspective of the users, not from the perspective of the data curators

Integrating multi-omics data is a hard job, and several aspects should be taken into account. While you work on the integration of different data, coming from different data sources, perhaps having different formats and different origins, you might be tempted to see the whole project only from the point of view of the data curators: you and your colleagues working on it. But if you only consider the perspective of the data curators, the bioinformatics resource, when ready, would be eventually optimized only for data curators, and not for analysts and users who would take advantage of it. This would bring several drawbacks, making your bioinformatics data resource difficult to use for analysts and researchers, perhaps making it unnoticed or forgotten by the scientific community.

Instead, we suggest always keeping in mind the perspective and the point of view of the users and the analysis who would ultimately exploit the integrated bioinformatics data resource. Of course, we know that it is easier to write it than to do it, but this aspect can be the single most important aspect of a multi-omics data integration project, which can mean the difference between failure and success.

We therefore recommend designing some real use case scenarios in which users can exploit the bioinformatics data resource to solve a real scientific problem. Pretend you are the analyst who needs to solve a particular biomedical problem and would like to use your bioinformatics resource. What would you need? What is missing? What is difficult to do? What could be improved? Answering these questions precisely and thoroughly would help you make a better, improved multi-omics integrated resource.

ENCODE [14] is a great example of a popular, documented, and useful multi-omics data integration project designed from the perspective of the users. Moreover, we also suggest applying existing integrative methods to the data to get the full user experience.

### Tip 2: Preprocess your data: Standardise and harmonise it

Standardising raw data helps to ensure that data from different omics technologies are compatible since they all have their own specific characteristics (for example, different measurement units, etc.). This process can involve a variety of different steps, such as normalizing data to account for differences in sample size or concentration, converting data to a common scale or unit of measurement, removing technical biases or artifacts, and filtering data to remove outliers or low-quality data points.

For small- and medium-scale studies, storing the raw data is important to ensure the full reproducibility of the results [15]. Giving access to the raw instrumentation data mitigates the issue that processing steps may vary, and allows researchers to make preprocessing assumptions that are appropriate for the selected downstream analysis.

When collecting multi-omics data, it is important to consider a sample size that can provide enough statistical power, and generate replicates, documentation, and project metadata, together with proper data management practices. In addition, the data need to be collected in a way that removes any possible sampling bias [16]. For preprocessed data, it is good practice to include full descriptions of the samples, equipment, and software used.

Data formats of multi-omics can vary widely, even within the same study. Therefore, for compatibility with machine learning or statistical analysis methods, further processing is often needed to unify the format, for example, n-by-k samples-by-feature matrix. This often needs also standard steps like normalization and batch effect correction [17,18].

Standardization and harmonization of data and metadata are key steps in multi-omics data integration because they help to ensure that data can be accurately and consistently interpreted and analyzed.

Standardization refers to the process of ensuring that data are collected, processed, and stored in a consistent manner, using agreed-upon standards and protocols. Lots of tools for standardising omics data have been developed over the last decade [9,19–21] in order to make the data comparable across different studies and platforms, in addition to make it easier to integrate and analyze data from multiple sources.

On the other hand, harmonization refers to the process of aligning data from different sources so that they can be integrated and analyzed together. This typically involves mapping data from different sources onto a common scale or reference and may involve the use of domain-specific ontologies or other standardized data formats [22–25]. Nikolai Russkikh and coauthors [26], for example, employed a style transfer method based on conditional variational autoencoders for RNA-seq data harmonization.

Moreover, it is important to describe precisely the preprocessing and normalisation techniques used in the project documentation and in the article associated with the project. This information would usually be inserted in the supplementary material of a scientific paper. If you have the authorization to release the data, we recommend releasing both the raw data and the preprocessed data in public repositories (Tip 8). Some users, in fact, might be interested in analyzing the raw data, depending on the aim of their projects.

### Tip 3: Value your data with metadata

Metadata are simply data that describe the main data. When a photographer takes a photo with a modern camera, for example, the camera not only saves the photo itself, but also records additional details such as lenses used, time and date at which the picture was taken, focal length, image resolution, and color profiles [27]. All these data are the metadata of that photo. These metadata are not the photo, but rather describe the photo, and they can facilitate image processing, image search, and image retrieval [27]. Of course, the photo is still the main protagonist of photography, but the role of the photo’s metadata is pivotal: They are the first pieces of the documentation of that photo and will be used by the photographer in several ways. If the metadata were absent, it would be complicated and almost impossible for the photographer to use it. Therefore, metadata are as important as data, not only in photography but in any field. Also in bioinformatics data integration, of course, metadata have an extremely important role. We therefore recommend paying particular attention to the curation of the metadata [28]. Any relevant information regarding a data element should be recorded in the metadata.

Examples of bioinformatics metadata can be found in multiple datasets. The GSE45255 dataset, for example, contains microarray gene expression of samples of patients with breast tumors [29,30] and is available on Gene Expression Omnibus (GEO). In bash, this dataset can be found through the software package geoCancerPrognosticDatasetsRetriever [31] and in R it can be downloaded through geneExpressionFromGEO [32].

This public dataset contains not only the gene expression data of the patients’ samples, but also relevant information about each patient, recorded in metadata [30]: lymph node status, estrogen receptors status, progesterone receptor status, human epidermal growth factor receptor 2 (HER2) status, histological grade, size in millimetres, adjuvant treatment, chemotherapy, recurrence or death from breast cancer, distant metastasis or death from breast cancer, and death from breast cancer. Of course, these metadata help researchers perform better scientific analyses on these datasets, allowing them to make additional discoveries regarding this dataset and therefore regarding breast cancer.

Moreover, ready-to-use data might contain outliers or unexpected data instances [33], and in those cases, the availability of metadata can be necessary to understand what actually happened in the generation of those data elements. Several approaches can be used to include metadata in bioinformatics data [34]; an interesting technique is to use data of different types from the principle dataset. For example, in metabolomics [35], Oliver Fiehn and colleagues [36] added mass spectrometry metadata to the physiological, clinical, and genomic data. Some bioinformatics teams developed structured metadata management tools, such as BioSamples [37] and medna-metadata [38].

Regarding metadata curation and its importance, it is relevant to mention the initiatives of Susanna-Assunta Sansone, a researcher who has advocated for the standardization and the structured curation of metadata for several decades through multiple resources: FAIRsharing [39], Collaborative Open Plant Omics (COPO) [40], Investigation/Study/Assay (ISA) Metadata Framework [41], and machine-actionable metadata models [42], just to mention a few.

### Tip 4: Take into account the genomic coordinates of the data, and not only the biological concepts

The integration of omics data is the process of combining data from different omics technologies (such as genomics, transcriptomics, proteomics, and metabolomics) to gain a more comprehensive understanding of a biological system. It is a powerful approach that can provide insights into the molecular mechanisms underlying complex diseases, identify potential therapeutic targets, and improve our understanding of fundamental biological processes.

We therefore recommend not to focus only on the anatomical sites of your samples or the disease that affects the host and always take into account the genomic regions of your data [43–46]. We refer to anatomical sites, tissues, organs, and diseases as biological concepts in this study. Considering the genomic regions helps in the identification of specific mechanisms underlying the regulation of gene expression and protein function, which can be crucial for understanding the molecular basis of diseases and identifying potential therapeutic targets.

Always use genome annotation tools that allow mapping your data onto specific genomic regions [43,47–49]. However, it is important to be careful when selecting genome annotation tools, as the choice of reference genome can have a significant impact on the results of your analyses. Different versions of the reference genome can have different sets of annotated features, such as genes, regulatory elements, and structural variations, and these differences can affect the interpretation of your data. For example, if you use a newer version of the reference genome that includes additional annotated features, you may identify different sets of genes or regulatory elements as being differentially expressed or altered in your samples. Therefore, it is important to carefully consider the version of the reference genome that you use when selecting genome annotation tools. In general, it is recommended to use the most up-to-date version of the reference genome that is available, as this will provide the most complete and accurate set of annotated features. However, it is also important to ensure that the version of the reference genome is appropriate for your data.

### Tip 5: Control for molecular signal redundancy via variable selection

The process of integrating data from different omic sources, commonly referred to as data fusion, has two major advantages: It preserves the original features of the data, and its flexibility allows for the mixing of data from multiple sources. However, omics signal redundancy needs to be carefully taken into account [3]. For instance, transcriptomics and proteomics are often (weakly) correlated [50], and methods that do not take into account (and filter appropriately) cross-omics information may perform the integration based on redundant signal. Normally, the redundancy depends on the organism being considered, and therefore the level of correction needs to be considered at an organism-specific level.

One solution is reducing the number of variables in an omic-specific or cross-modal way, which generates a more usable representation. With the advent of machine learning tools, supervised techniques can perform such filtering if appropriate clinical/output labels are available, for example, with a feature importance analysis. In general, selecting features decreases the level of noise and can be used as a method to balance the number of features across the omics. Furthermore, this also reduces the risk of overfitting. In the presence of a large dataset, it is also worth considering whether the redundancy can be addressed a priori, for instance, using a clinical stratification approach [51].

Variable selection can also be performed with unsupervised techniques, in a single-modal or multi-modal fashion, with tools like MOFA [52], JIVE [53], and sparse PLS [54]. Acharjee and colleagues [55], for example, took advantage of retention time-dependent clustering to remove the signal redundancy from the metabolite data involved in the omics integration. Similarly, Cao and Gao [56] recently proposed an integration method to mitigate the redundancy in single-cell data by explicitly modelling the regulatory interactions taking place across the omics layers.

### Tip 6: Try different integration approaches

When integrating multi-omics data via machine learning models, it is important to note that the technique to be adopted depends on the dataset and the task at hand and cannot usually be decided a priori [57]. A simple concatenation of features across the omics (*early integration*) is a viable approach but is likely to generate enormous matrices, outliers, highly correlated variables, noise, and other difficulties.

*Intermediate integration* is a viable alternative, in which the idea is to jointly integrate the features across the omics without prior omic-specific processing. The advantage of this approach is the possibility to process the features based on their redundancy or complementarity both within each omic and across the different omics [58]. Intermediate integration is based on the assumption that all the omics can be mapped onto a shared latent space. Therefore, disparities among the omics may bring challenges and lead to an imbalanced learning process. Furthermore, it often depends on unsupervised matrix factorization, which has difficulty incorporating substantial amounts of preexisting biological knowledge.

*Late integration* is another option, often based on ensemble machine learning methods. Specifically, a model is first trained for each omic to perform the prediction independently, and then the predictions achieved from each omic are combined via averaging or voting. Late integration may be appropriate when the predictive performance is unbalanced across the omics, for instance, if one omic is significantly more predictive than others, but the integration with the other omics still improves the overall performance. However, it does not directly integrate the data and may overlook cross-omics relationships [59]. Finally, hierarchical approaches or mixed integration strategies are also potential alternatives [60].

### Tip 7: Prepare your data for multi-omics data integration with machine learning

Several supervised and unsupervised machine learning techniques have been successfully employed for multi-omic data analysis and integration [61]. Supervised learning trains a model by using preassigned labels for each sample, for instance, the subtype of a given cancer (classification problem) or the overall survival probability of a patient (regression problem). The model is trained using the data at hand, but in a way that does not overfit the same data, as the goal is then to use the trained model for achieving accurate predictions when new or “unseen” samples are considered. Conversely, unsupervised learning can be applied when labels are not available and can be used to reduce the dimensionality of the dataset or detect patterns or clusters within the samples. However, in complex phenotypes like cancer, where the interaction between events spanning multiple omics layers is likely to be the main cause of progression, traditional data-driven multi-omics methods based on machine learning are only able to uncover associations among genes, proteins, or other omics components, without offering a mechanistic interpretation [62].

In this regard, it is possible to use systems biology techniques for omics data integration and to provide further mechanistic knowledge to be incorporated into machine learning approaches. While recognising the role of individual components within a biological system, systems biology follows the notion that “the whole is greater than the sum of its parts.” Specifically, it aims at investigating a biological system as a whole and with an integrated approach, namely by considering the interactions between different components as a way to enrich and explain the behaviour of each component [63].

Among such tools, genome-scale metabolic models (GSMMs) are mathematical reconstructions of metabolic networks that can be used as scaffolds for further omics data integration, therefore generating patient- or condition-specific models that achieve more accurate predictions of disease phenotypes [25,64–66].

In the machine learning era, the effective integration of data-driven and knowledge-driven approaches is increasingly being recognized as key to improving the outcome of omics integration studies, for example, biomarker prediction or phenotype characterisation [67]. For instance, adding features derived or processed through modelling techniques can incorporate knowledge into the model and allow omics data interpretation on a mechanistic or phenotypic level, rather than merely on a data-driven level [68,69].

To ensure the biological interpretability of the results, it is important to focus on methodological advances that can combine multi-omics integration and knowledge extraction with modelling techniques [70,71]. Importantly, since omics data can be quantified numerically and in a condition-, tissue-, and patient-specific way (for example, transcriptomic profiles, protein levels, and metabolite concentrations), using such models within machine learning pipelines can filter out some of the redundancy inherently present in genome-scale omics data. The long-term goal is to find the trade-off between selecting features via machine learning or data-driven approaches only (which has no biological rationale), and using biology-informed approaches (which is likely to lead to suboptimal machine learning performance) [72,73].

### Tip 8: Use open science best practices

When starting a new computational biology project, one often has the possibility to decide which programming languages, software platforms, and data query languages to use. Similarly to what we recommended for machine learning [74], pathway enrichment analysis [75], data cleaning and feature engineering [33], and medical image analysis [76], we advocate for using only open-source computer languages and software programs.

Open-source programming languages (such as R or Python), open-source software platforms (Bioconductor [77], Bioconda [78] and Galaxy [79], Anvi’o [80]), open-source data query languages (SQL), and open-source relational database management systems (PostgreSQL and SQLite) can bring several advantages to your multi-omics project, compared to proprietary software.

Open-source material, in fact, can be shared easily among colleagues and collaborators, without worrying about licenses. Moreover, open-source technology can be updated and upgraded easily and most of the time free of cost and can be reutilized in several other projects. If one needs to switch labs, institutes, or jobs, one can take their software and code with themselves in the new environment.

For operating systems, we suggest Linux Ubuntu; for distributed systems, we recommend Apache Spark; for office productivity software, we advocate for LibreOffice. Regarding software code sharing, we advise sharing your code online openly on public epositories such GitHub, GitLab, and Bitbucket, for example. You can take all the scripts that you developed for your multi-omics integration and release them publicly on the internet. This practice would allow the reproducibility of your work and let other users around the world find possible mistakes in your analyses, allowing you to correct them and ultimately generate better, more solid results.

Once one has spent several months and energy working on a multi-omics integrated data resource, of course we suggest releasing it publicly online, following the findability, accessibility, interoperability, and reusability (FAIR) principles [81]. A researcher can release their data on free open platforms such as Kaggle [82], University of California Irvine Machine Learning Repository [83], FigShare [84], or Zenodo [85]. One can also consider creating and releasing their own data repository [86,87].

Releasing data online would permit other researchers in the world to analyze them and therefore to make new scientific discoveries through secondary analyses [88,89]. The more available the data are, the more secondary studies will be carried out, the higher impact a dataset can have, also in terms of article citations.

Regarding the publication, once the study manuscript is ready for submission to a scientific journal, we suggest releasing it as a preprint on bioRxiv, medRxiv, or arXiv. Moreover, if one has the chance to choose which scientific journals to submit their articles to, we advocate for open-access journals. Open-access articles, in fact, can be freely read and accessed by anyone in the world, including high school students and researchers from developing countries. Open-access journals can be found on the Scimago Journal Ranking website [90].

### Tip 9: Use ad hoc software libraries and query languages, do not develop new scripts on your own

There are several reasons why you might consider using ad hoc software libraries and query languages for integrating omics data than developing new scripts on your own:

1. Time and resources: developing your own scripts for integrating omics data can be a time-consuming and resource-intensive process, especially if you are not familiar with the specific programming languages and tools that are commonly used in the bioinformatics domain;
2. Accuracy and reliability: ad hoc software libraries and query languages are usually developed and maintained by experts in the field and have been extensively tested and validated. This means that you can usually be confident in the accuracy and reliability of the results produced by these tools;
3. Community support: when you use consolidated tools, you can benefit from the support of a larger community of users who are familiar with the tools and can provide guidance and help if you face any issues. This can be extremely helpful especially if you are new to these tools;
4. Compatibility with other tools: ad hoc software libraries and query languages are often designed to be compatible with other tools and resources in the field. This can make it easier to integrate your results with other data sources or to use the tools in conjunction with other software;
5. Easy of use: ad hoc software libraries and query languages are often designed with usability in mind and may offer user-friendly interfaces and documentation to help you get started. This can make it easier for you to use the tools, even if you are not an experienced programmer.

Overall, keep in mind the aforementioned points before writing your own scripts. There are tons of open-source libraries and tools for roughly every kind of omics data integration and analysis distributed over public repositories like the Python Package Index [91] and Bioconductor [77] with Python and R packages, respectively, as well as Bioconda [78] and the Galaxy ToolShed [92], which extremely simplify the process of distributing and effectively using such kind of software tools for managing, integrating, and analysing multi-omics data. These factors can make it more efficient and effective for you to integrate omics data and can help to ensure the quality and reliability of your results.

### Tip 10: Document everything

We already mentioned the importance of metadata, data that describe the principle data. Metadata can also be seen as a structured form of documentation.

In scientific research, well-written documentation and reporting are as important as the scientific discoveries themselves [93]. Also in software development, well-written documentation is as important as the software itself [94].

Documentation should be as detailed as possible: addressed both to developers who want to redo the integration analysis and to users that just want to learn more about the whole bioinformatics process.

The same rule for scientific research and software development is true for bioinformatics data integration: document everything. Document how you obtained the data, how you integrated them and why, which data sources you selected and why, which technologies you decided to use and why, and so on. This documentation will be invaluable for your colleagues and collaborators, but also for your future self that will need to recover some information about the data integration resource. The documentation will also be pivotal for writing the article on the study.

The documentation should be written following some precise standards [95] and not only at the end of the project, but during its development. Ongoing documentation should be recorded in a notebook or scientific diary [96]. Good examples of documentation can be found on the Bioconductor website: for example, the tutorial for the usage of OMICsPCA R software package [97].

## Conclusions

Integration of multi-omics data is a key aspect of bioinformatics, since assembling data from different sources can surely enrich the scientific description of a phenomenon. Integrated data derived from different sources can then be used for computational analysis through machine learning or biostatistics methods and eventually lead to better, more solid results and outcomes. Multi-omics data integration, however, can suffer from many pitfalls and common mistakes, which sometimes might even go unnoticed, and which might undermine or even corrupt the final scientific results of the analysis.

To prevent these common mistakes, we present here our ten quick tips for multi-omics data integration, which we designed for any data curator, biomedical data scientist, machine learning analyst, computational biologist, bioinformatician, and student who wants to perform these steps to generate an omics data resource. We believe that our simple guidelines, if followed correctly, can improve the quality of the multi-omics data integration phase and therefore help generate better results, which can help us better understand the underlying biology of the system being studied.
